# Genotype-phenotype associations in microtia: a systematic review

**DOI:** 10.1186/s13023-024-03142-9

**Published:** 2024-04-09

**Authors:** Siti Isya Wahdini, Fina Idamatussilmi, Rachmaniar Pramanasari, Almas Nur Prawoto, Citrawati Dyah Kencono Wungu, Indri Lakhsmi Putri, Gunadi

**Affiliations:** 1 Plastic Reconstructive and Aesthetic Surgery Division, Department of Surgery, Faculty of Medicine, Public Health and Nursing, Universitas Gadjah Mada /Dr. Sardjito Hospital, Yogyakarta, Indonesia; 2 Plastic Reconstructive and Aesthetic Surgery Department, Faculty of Medicine, Airlangga University/Airlangga University Hospital, Surabaya, East Java Indonesia; 3 Department of Physiology and Medical Biochemistry, Faculty of Medicine, Airlangga University, Surabaya, East Java Indonesia; 4 Pediatric Surgery Division, Department of Surgery, Genetics Working Group/Translational Research Unit, Faculty of Medicine, Public Health and Nursing, Universitas Gadjah Mada/Dr. Sardjito Hospital, Jl. Kesehatan No. 1, Yogyakarta, 55281 Indonesia

**Keywords:** Microtia, Syndromic, Non-syndromic, Genotype- phenotype association, *TCOF1*, *SIX2*, *HSPA9*, *GSC* exon 2, *FANCB*, *HOXA2*, *GSC* exon 3, *MARS*, *CDT1*

## Abstract

**Background:**

Microtia is a congenital ear malformation that can occur as isolated microtia or as part of a syndrome. The etiology is currently poorly understood, although there is strong evidence that genetics has a role in the occurrence of microtia. This systematic review aimed to determine the genes involved and the abnormalities in microtia patients' head and neck regions.

**Methods:**

We used seven search engines to search all known literature on the genetic and phenotypic variables associated with the development or outcome of microtia. The identified publications were screened and selected based on inclusion and exclusion criteria and assessed for methodological quality using the Joanna Briggs Institute (JBI) critical appraisal tools. We found 40 papers in this systematic review with phenotypic data in microtia involving 1459 patients and 30 articles containing genetic data involved in microtia.

**Result:**

The most common accompanying phenotype of all microtia patients was external ear canal atresia, while the most common head and neck abnormalities were the auricular, mental, and oral regions. The most common syndrome found was craniofacial microsomia syndrome. In the syndromic microtia group, the most common genes were *TCOF1* (43.75%), *SIX2* (4.69%), and *HSPA9* (4.69%), while in the non-syndromic microtia group, the most frequently found gene was *GSC* exon 2 (25%), *FANCB* (16.67%), *HOXA2* (8.33%), *GSC* exon 3 (8.33%), *MARS1* (8.33%), and *CDT1* (8.33%).

**Conclusions:**

Our systematic review shows some genes involved in the microtia development, including *TCOF1, SIX2, HSPA9, GSC* exon 2*, FANCB, HOXA2, GSC exon 3, MARS1,* and *CDT1* genes. We also reveal a genotype-phenotype association in microtia. In addition, further studies with more complete and comprehensive data are needed, including patients with complete data on syndromes, phenotypes, and genotypes.

**Supplementary Information:**

The online version contains supplementary material available at 10.1186/s13023-024-03142-9.

## Background

Microtia is a congenital malformation of the ear with varying degrees of severity, ranging from mild structural problems to a completely missing external ear. In current literature, microtia could also be called anotia, small ear, or ear deformity [1].

The presentation of microtia includes minimal morphological abnormalities to the complete absence of the ear. Microtia can occur as the only clinical abnormality referred to as isolated microtia or with other associated anomalies as part of a syndrome that is referred to as syndromic microtia, which present with other congenital facial anomalies due to abnormal development or growth of associated embryological structures [2].

Numerous syndromes have been associated with microtia, including Treacher-Collins Syndrome (TCS, MIM #154500), craniofacial/hemifacial microsomia (CFM, MIM #164210), Goldenhar Syndrome (MIM #164210), Nager Syndrome/Acrofacial Dysostosis (AFD, MIM #154400), Crouzon Syndrome (MIM #123500), Apert Syndrome (MIM #101200), and Klippel-Feil Syndrome (KFS, MIM #118100) or Wildervanck Syndrome (MIM #314600). The classification of syndromic microtia is based on the constellation of clinical features and the underlying genetic or environmental etiology [2]. In the current literature, microtia is classified as part of the Oculo-Auriculo-Vertebral Spectrum (OAVS, MIM #164210**)**. Associations between microtia and other features included in OAVS are said to have overlapping phenotypes [3].

In addition, there is a similar general etiological basis, in which there are malformations of structures derived from first and second branchial arches, including eyes, mouth (lips, tongue, and palate), ear, maxilla, and mandible. Various classification systems to define definite feature criteria associated with OAVS have been proposed, such as the OMENS classification (Orbit, Mandible, Ear, Nerve, and Soft Tissue). However, consensus on the minimum diagnostic criteria for OAVS is still limited and has led to the controversial concept that most (or all) cases presenting with isolated microtia are also referred to as OAVS, which should be considered separate entities. Nevertheless, there is an overlapping clinical expression in microtia and OAVS, and many common underlying genetic disorders may exist [3].

The etiology of microtia has contributions from both genetic and non-genetic components. Prenatal alcohol exposure in the mother, retinoids, or diabetes in the mother were thought to be environmental factors. The existence of a genetic contribution to microtia is supported by various evidence, such as identifying families with variable expression and incomplete penetration that are separated as autosomal dominant, autosomal recessive, or multifactorial traits. In addition, there was greater concordance between monozygotic versus dizygotic twins (38.5% vs 4.5%, respectively). There are also differences in prevalence between ethnicities, such as Hispanics (1.12/10,000), US-born Hispanics (0.83/10,000), Asian (0.54/10,000), native Pacific Islanders (4.61/10,000), and the Philippines (4.77/10,000) population. In microtia developed in murine models, genetic mutations were identified in several microtia patients, and more than 50 chromosomal and monogenic syndromes were observed in microtia in the clinical spectrum [4].

In 1926, Marx classified microtia into three grades: 1) abnormal auricle with all identifiable landmarks, (2) abnormal auricle without some identifiable landmarks, and 3) tiny auricular tag or anotia. Rogers proposed a fourth-grade classification, with grade IV being anotia. Other classifications were then developed by Tanzer in 1978, Weerda in 1988, and Hunter et al. in 2009, who classified it into 1) microtia, first degree: the presence of all the standard ear components and the median longitudinal length of more than 2 SD below the mean; 2) microtia, second degree: median longitudinal length of the ear more than 2 SD below the mean in the presence of some but not all, parts of the normal ear; 3) microtia, third degree: the presence of some auricular structures, but none of these structures conforms to recognized ear components; and 4) anotia, where complete absence of the ear is found [3].

Based on the etiological subtype, microtia can be classified into: 1) monogenic form, namely microtia attributed to mutations or alterations in a single gene (*HOXA* and *HOXD* gene clusters, *TCOF1*, *POLR1C, POLR1D,* and *GLI3*) [4], another study identified candidate genetic variants for microtia, such as the *HOX (HOXA1/HOXB1/HOXA2), SIX, EYA,* and *TBX1* [5]; 2) chromosomal aberrations, when chromosomal abnormalities occur, such as deletions, duplications, or rearrangements. For example, deletions in chromosome 22q11.2 are associated with DiGeorge syndrome, which can present with microtia as part of its phenotypic spectrum; 3) teratogenic causes are exposure to teratogenic agents during critical periods of embryonic development. Maternal use of certain medications, infections, or exposure to environmental toxins such as alcohol or retinoic acid has been linked to microtia; 4) sporadic/multifactorial form is without a clear underlying genetic or environmental cause. These forms are likely multifactorial, involving a combination of genetic susceptibility and environmental factors; the exact contributions of individual genes or environmental influences are often difficult to discern in these cases. However, despite these findings, the etiology underlying microtia in most patients is still not fully understood [6].

The etiology of microtia, either isolated or associated with other syndromes, is still poorly understood. There is strong evidence that genetics has a role in the occurrence of microtia. Although several studies have identified candidate genetic variants for microtia, no causal or potential genetic mutations have been confirmed.

Based on current data, the most common abnormalities are in the head and neck region, ophthalmologic abnormalities, and kidney malformations consecutively [7], with the most frequent simultaneous dysmorphic features with microtia including cleft palate, cleft lip and palate, anophthalmia/microphthalmia, facial asymmetry, and macrostomia [8]. Hence, the etiology and prevalence of related malformations with microtia are still unclear due to multifactorial causes such as maternal nutritional deficiencies, drug-related disease during pregnancies, alcoholism, carcinogenic exposure, and blockage to the blood supply due to pressure from the positioning of the fetus. Therefore, based on current literature, this study aimed to determine the genes involved and the abnormalities present in microtia patients' head and neck regions.

## Materials and methods

### Protocol and registration

The International Prospective Register of Systematic Reviews (PROSPERO, CRD42022340150 (28/06/22)) has received our protocol. PROSPERO was also examined for similar systematic reviews. No approved methodology looked into the genetic causes of microtia. This systematic review report followed the Preferred Reporting Items for Systematic Review and Meta-Analysis (PRISMA) standards [9].

### Eligibility criteria

We thoroughly reviewed all known research on the genetic and phenotypic variables associated with the development or outcome of microtia. We aimed to present systematic evidence regarding the genotype and phenotype in the head and neck associated with microtia. First, the titles and abstracts of the identified publications are evaluated for relevance to the topic of interest. Using Mendeley, the identified papers were checked for duplication. The full text of all screened articles was then analyzed for inclusion criteria, including observational studies of case-control, cohort, and case reports/series in English evaluating genetic or phenotypic variables in microtia. Animal studies, textbooks, conferences, guidelines, correspondence, not available full text, duplications, literature reviews, systematic reviews and meta-analysis, and articles that lack information about genotype and phenotype in Microtia were excluded.

### Search strategy

The search and study selection was carried out by five writers (S.I.W., F.I., R.P, ANP, and ILP), who were overseen by the two authors (CDKW and G.) from April until June 2022. We used seven electronic bibliographic databases: EBSCO EDLINE, ProQuest, PubMed, Sage Journal, Science Direct, Scopus, and Wiley. The details of search keywords are listed in Supplementary file 1.

### Data extraction

Seven reviewers (S.I.W., F.I., R.P, A.P., I.L.P., C.D.K.W, and G.) worked separately to extract data using a standardized form. The Joanna Briggs Institute (JBI) critical assessment methods were used to assess the methodological quality of the research in this systematic review [10].

Four reviewers (S.I.W, F.I., R.P, and A.P.) extracted relevant data about study characteristics (study design, area of origin, year of publication, and the number of patients) using predefined forms. The characteristics data of the patients extracted were gender, age, and syndrome. The phenotype data we extracted was the grade, affected side of the ear with microtia, and other phenotypes in the patient's head and neck.

We extracted the genes involved with microtia, characteristics of the mutated genes, levels of genetic abnormality, type of gene mutation, sequencing system, and outcome data. Then, the differences of opinion between the two reviewers were resolved by discussing with four reviewers. S.I.W. and I.L.P. regarding the phenotypes section, and C.D.K.W. and G. regarding the genetics section.

## Results

### Systematic review outline

**Fig. 1:**
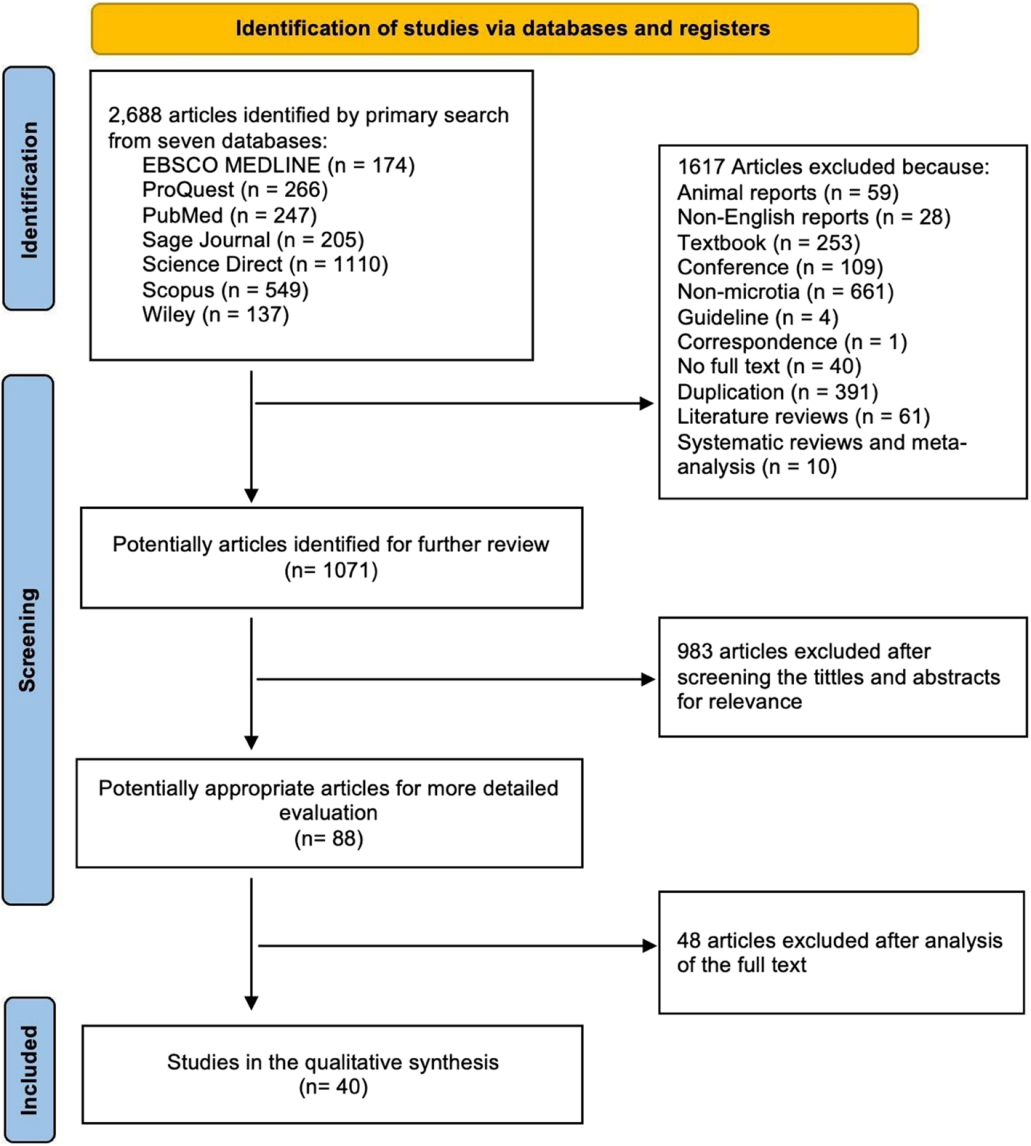
PRISMA flow diagram of the study inclusion process

A total of 1071 articles were evaluated for inclusion in this systematic review, and then 983 articles were excluded based on the relevance of the title and abstract review. Eighty-eight full-text articles were further analyzed for eligibility, and 40 articles were finally included (Fig. 1). The eligible studies were evaluated using a checklist questions form provided by JBI tools based on the methodology of the investigations. All publications implicated were rated as low-risk bias using the JBI Tools for case reports, case series, cohort, and case controls (see Supplementary file 2).

### Study characteristics

**Fig. 2:**
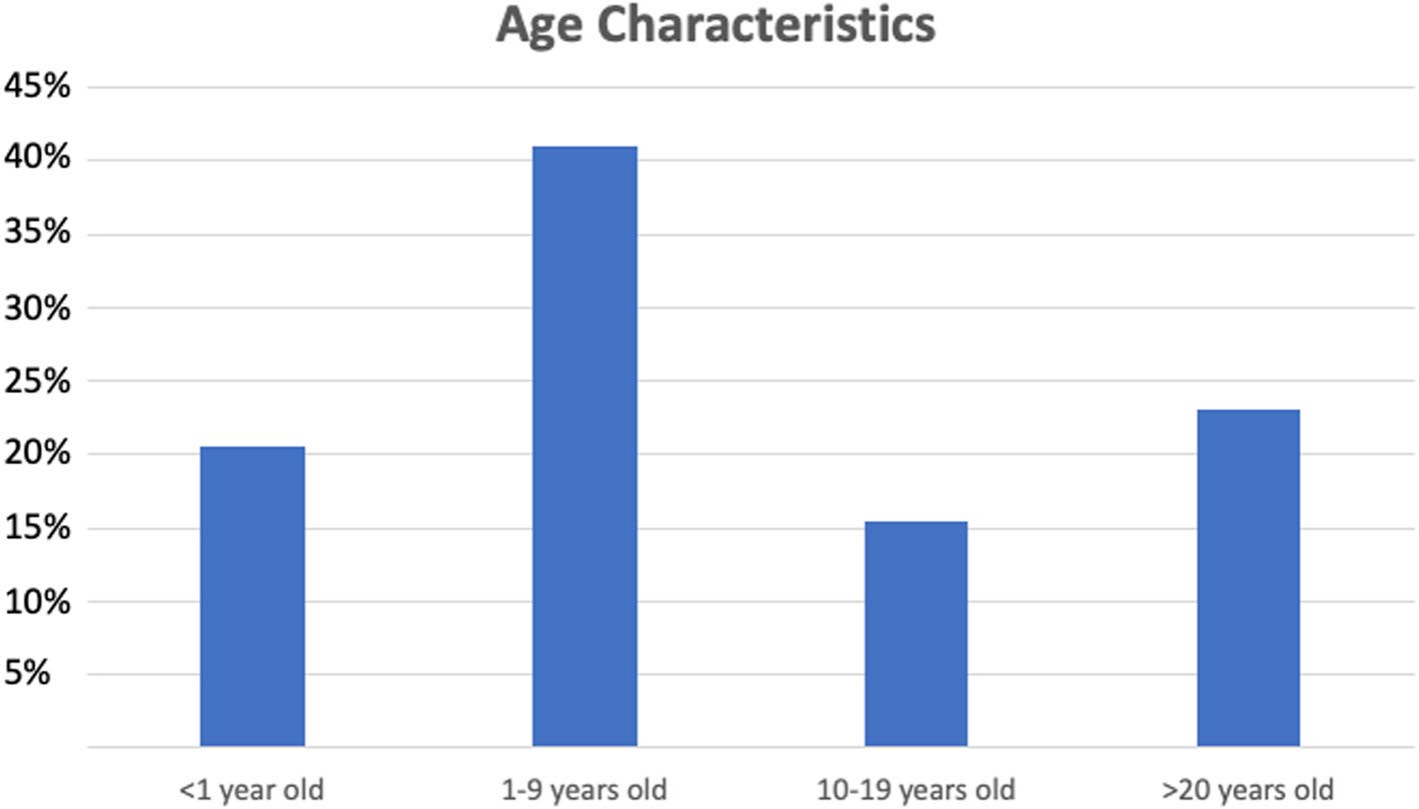
Patient age characteristics (*n=* 39)

We found 40 papers in this systematic review: 40 papers containing phenotypic data in microtia involving 1459 patients (Table 1) and 30 articles containing gene data involved in microtia (Table 2). A total of 1459 cases were obtained, of which 1,193 cases were unreported gender, 186 males and 80 females (Table 3). From 1459 patients with microtia phenotype, we found the most age range was 1-9 years old (41%), followed by age > 20 years old (23%) (Fig. 2).

**Table 1:** Characteristics of the studies of phenotypes in microtia

**No.**	**First author’s surname/country of origin/year of publication**	**Study design**	**Sample size**	**Sex**	**Syndrome**	**Affected ear**	**Grade**	**Other Head & Neck Phenotypes**
1.	Gimelli/Italy/2013 [11]	CR	1	F	TCS, CFM	UNI - R	-	External auditory canal stenosis, CT-Scan: aplasia cranial fossa floor, reduced tympanic volume, Dysplasia of Malleus incudal and Stapes-incudal articulations
2.	Glaeser/Brazil/2021 [12]	CR	1	F	CES, OAVS, CFM	UNI - L	-	External auditory canal atresia, Preauricular tags, Hypertelorism, downward slanting palpebral fissures, epicanthic folds, cerebral hypoplasia, ventricular dilatation
3.	Tassano/Italy/2015 [13]	CR	1	F	-	UNI -L	-	External auditory canal atresia, cleft palate, SNHL, CT-Scan: reduced tympanic volume, Dysplasia of Malleus incudal and Stapes-incudal articulations, Hypoplasia of mastoid complex
4.	Chaves/Brazil/2019 [14]	CR	1	M	-	-	-	High-arched palate, Dolichocephaly, Narrow face, intellectual disability
5.	Huang/China/2013 [15]	CR	1	F	CFM	UNI -L	-	Anophthalmia, orbital hypoplasia, eyelid coloboma, preauricular tags, Cheek / soft tissue tags, Mandibular hypoplasia, cleft lip & palate, macrostomia, hypertelorism, CT Scan: Zygoma/malar hypoplasia, maxillary hypoplasia, Incomplete Closure of the anterior fontanelle, Fissure in the alveolar crest, Forehead retrusion
6.	Kim/South Korea/2020 [16]	CR	1	M	MFDM	-	-	Low set ears, micrognathia, microcephaly, Mixed Hearing loss
7.	Goldmuntz/Philadelphia/2011 [17]	CR	1	-	CFM	UNI -R	-	Posterior embryotoxon, high-arched palate, epicanthic folds, tented lip,
8.	Brun/France/2012 [18]	CR	1	M	15q24 deletion Syndrome, OAVS, CFM	UNI -R	IV	Mandibular hypoplasia, High anterior hairline, Broad Medial eyebrow, Bulbous nose, Zygoma / malar hypoplasia, CHL, ID
9.	Koprulu/Turkey/2021 [19]	CR	1	M	Fraser Syndrome	-	I	Hypertrophied frontal & maxillary sinuses, underdeveloped supraorbital ridge, prominent maxilla with overbite and malocclusion,perincisors are overjet, retrognathia,diastema, dental crowding, and unerupted teeth,
10.	Hu/America/2019 [20]	CR	1	-	-	-	-	Cleft lip & palate, semi lobar HPE, Corpus Callosum Dysgenesis
11.	Jarzabek/Poland/2012 [21]	CR	1	M	Kallmann Syndrome	UNI - R	-	Preauricular tags, cleft lip & palate, Zygoma/Malar hypoplasia, Prognathism, Exophthalmia, P rominent Nasal Bone,
12.	DeGolovine/Texas/2012 [22]	CR	1	F	Goldenhar Syndrome, CFM	UNI -L	-	External auditory canal stenosis,preauricular tags, Hyper-segmented Cervical Vertebrae (C2-C3), Downward Slant of mouth side
13.	Griffith/Indianapolis/2009 [23]	CR	1	M	Trisomy 13 mosaicism	UNI - R	-	High-arched palate, epicanthic folds, Bulbous nose, Sloping forehead with a telangiectatic nevus, Deep-set eyes, Thin Upper Lip
14.	Knapp/Maryland/2020 [24]	CR	1	M	MGORS	UNI - R	-	Cleft palate, Optic Nerve hypoplasia, Bifid Uvula
15.	Saviola/Italy/2021 [25]	CR	1	M	Coffin- Siris Syndrome	UNI - R	-	Corpus Callosum Dysgenesis, Hashimoto Thyroiditis
16.	Lacour/New Orleans/2018 [26]	CR	1	M	MFDM, CFM	BIL	RI/LIII	External auditory canal atresia, micrognathia, Zygoma/Malar hypoplasia, Dysplastic Ear, Etopic craniosynostosis, Trigonocephaly
17.	Lalani/Texas/2019 [27]	CR	1	-	-	-	-	Low set ear, micrognathia, speech delayed
18.	Bragagnolo/Brazil/2016 [28]	CR	1	F	WHS	UNI - L	-	Dermoid, preauricular tags, cleft palate, High Forehead, Tracheal Stenosis & malacia, Frontal Periventricular Gliosis
19.	Knapp/New Zealand/2020 [29]	CR	1	F	MGORS	UNI - L	-	Micrognathia, Prominent Nose, Full Bottom Lip
20.	Liu/China/2021 [30]	CR	1	M	TCS	UNI - L	-	Cup Ear deformity, mandibular hypoplasia, micrognathia, downward slanting palpebral fissures, Zygoma/Malar hypoplasia,
21.	Maya/Israel/2020 [31]	CR	1	F	Beals Syndrome	-	-	Micrognathia
22.	Okamoto/Japan/2022 [32]	CS	2	M:1 F: 1	-	-		Hypertelorism, low set ears, high-arched palate, micrognathia: 2 Microcephaly, SNHL : 1
23.	Brophy/Iowa USA/2013 [33]	CS	3	M: - F: -	BOR: 3	-	-	Ext. Audit. Canal atresia, cup ear deformity, untyped hearing loss: 2 Ext. Audit. Canal stenosis, preauricular pits, branchial fistula, branchial tag, smallmouth: 1
24.	Bukowska/Poland/2020 [34]	CS	3	M: 1 F: 2	TCS: 3	-	-	Mandibular hypoplasia, downward slanting palpebral fissures, maxillary hypoplasia, CHL: 3 Cleft & lip palate, absence of eyelid : 2
25.	Schmid/German/1985 [35]	CS	3	M: 2 F: 1	OAVS: 3	UNI: 3	-	External auditory canal atresia: 3 Right-sided palatoplegia: 2
								ysplasia M. Incudal and stapes-incudal articulations, granulomatous eardrum and the mastoid region, fistula of the ear: 1
26.	Sutphen/Texas/1995 [36]	CS	2	M: - F: 2	Goldenhar Syndrome: 2 CFM: 2	UNI: 2	I :1 III: 1	External auditory canal stenosis, mandibular hypoplasia: 2 Preauricular pits, macrostomia: 1
27.	Tingaud- Sequeira/France/2021 [37]	CS	2	M: 2 F: -	Goldenhar Syndrome: 2 CFM: 1	BIL: 2	III: 4	External auditory canal atresia, cleft palate, dysplastic ear: 2 Mandibular hypoplasia, zygoma/malar hypoplasia, maxillary hypoplasia: 1
28.	Kim/South Korea/2017 [38]	CS	2	M: 1 F: 1	-	-	-	External auditory canal stenosis, low set ears, high-arched palate, micrognathia, narrow nose, high nasal bridge, small mouth with full lips, microstomia: 2, Dysplastic ear, tracheal stenosis & malacia: 1
29.	Jung/New York/2020 [39]	CS	4	M: - F: -	-	-	-	Microcephaly, hydrocephalus, tracheoesophageal fistula: 3 External auditory canal stenosis, esophageal fistula, microphthalmia: 1
30.	Su/Taiwan/2007 [40]	CS	5	M: 2 F: 3	OAVS: 4 TCS: 1 CFM: 3	UNI: 4 BIL: 1	-	External auditory canal atresia: 4 Micrognathia, dysplasia of malleus incudal and stapes-incudal articulations, zygoma/malar hypoplasia: 3 Preauricular pits, mandibular hypoplasia, lateral oral cleft: 2 Cleft palate, downward slanting palpebral fissures, epicanthic folds, maxillary hypoplasia, dysplastic ear, multiple pinnae on the side, partial absence of eyelashes, ptosis: 1
31.	Martelli- Junior/Brazil/2009 [41]	CS	4	M: - F: -	TCS: 4	-	-	Eyelid coloboma, low set ears, downward slanting palpebral fissures, zygoma/malar hypoplasia, dysplastic ear, narrowed palate, partial absence of eyelashes,retrusive mandibular, facial implantation of the hair : 3 Narrowed frontal bone: 2 Anterior open bite: 1
32.	Chen/China/2018 [42]	CS	19	M:11 F: 8	TCS: 19		I: 8, II: 6 III: 4	Middle ear hypoplasia: 10; External auditory canal atresia: 11; CHL: 10; External auditory canal atresia: 8; Untyped hearing loss: 3; Mixed type hearing loss, cholesteatoma: 2; Cleft palate: 1
33.	Patton/London/1995 [43]	CS	5	M: 7 F: -	BOR: 5	UNI: 4 BIL: 1	-	External auditory canal atresia: 5 Cleft palate, dysplasia malleus incudal andstapes-incudal articulations, dysplastic ears: 1
34.	Royer- Bertrand/Switzerland/2015 [44]	CS	3	F: 3	EVEN-PLUS: 3	BIL: 3	II: 1 IV: 2	Flat nose, triangular nostrils, arched & synophrys eyebrows: 3 Aplasia cutis on the skull: 2 Persistent open anterior fontanelle, two lateral hair whorls, midface hypoplasia, brachycephaly, high palate,hypodontia,short neck: 1
35.	Heike/North America/2016 [45]	CH	91	-	CFM: 91	UNI: 80 BIL: 11	-	Dermoid: 7; Eyelid Coloboma: 2 ; External Auditory Canal Atresia: 36; Preauricular Tags: 22 ; Mandibular Hypoplasia: 46 ; Lateral Oral Cleft 8 ; Cleft Palate: 3 ; Cleft Lip Palate: 6
36.	Luquetti/US/2019 [46]	CH	103	-	CFM: 103	UNI: 72 BIL: 31	-	- (unclear)
37.	Zhang/China/2016 [47]	CH	984	-	CFM: 984	UNI: 1062 BIL: 42	II:241 III:727 IV: 16	- (unclear)
38.	Luquetti/US/2015 [48]	CC	70	M:49 F: 21	-			- (unclear)
39.	Monks/UK/2010 [7]	CC	12	M: 7 F: 5	-	UNI: 11 BIL: 1	II: 6 III: 3 IV: 3	- (isolated microtia)
40.	Zhang/China/2009 [49]	CC	121	-	-	-	-	- (isolated microtia)

*Abbreviations*: *TCS* Treacher-Collins Syndrome, *CFM* Craniofacial/hemifacial microsomia, *OAVS* Oculo-auriculo-vertebral spectrum, *MGORS* Meier- Gorlin Syndrome, *WHS* Wolf-Hirschhorn syndrome, *BOR* Branchio-oto-renal syndrome, *MFDM* Mandibulofacial dysostosis with microcephaly, *UNI* Unilateral, *R* Right, *L* Left, *F* Female, *M* Male, *SNHL* Sensorineural hearing loss, *CHL* Conductive hearing loss, *ID* Intellectual disability, *CR* Case report, *CS* Case series, *CH* Cohort, *CC* Case-control

**Table 2:** Characteristic of the studies of genetics involved in microtia

**No.**	**First author’s surname/country of origin/year of publication**	**Study design**	**Sample size**	**Syndrome**	**Related Gene**	**Genetic level of disorder**	**Mutation type – DNA / RNA Chromosome**	**Homozygous / Heterozygous**	**Inheritance**	**Sequencing System**
1.	Gimelli/Italy/ 2013 [50]	CR	1	TCS : 1	SPATA7 : 1	Chromosome	Interstitial deletion	Heterozygous	Autosomal Recessive	Array CGH
2.	Glaeser/Brazil/ 2021 [51]	CR	1	CES, OAVS, CFM	BCL2L13 : 1; BID : 1; CECR1 : 1; CECR2 : 1; CECR4 : 1; CECR5 : 1; CECR6 : 1; CECR7: 1; FLJ 41941 : 1; HSFY1P1 : 1; IL17RA: 1; MICAL3 : 1; MIR 3198 : 1; MIR648 : 1; PEX26 : 1; SLC25A18: 1; TUBA8e: 1; XKR3 : 1	Chromosome	Inverted duplication	Heterozygous	Mitochondrial	Whole Genome Array CGH
3.	Tassano/Italy/2015 [52]	CR	1	-	FOXI3 : 1	Chromosome	Interstitial deletion	Homozygous	Autosomal Dominant	PCR
4.	Chaves/Brazil/ 2019 [53]	CR	1	-	BBS4 : 1	DNA	Duplication	Homozygous	Autosomal Recessive	Microarray
5.	Huang/China/2013 [54]	CR	1	CFM	PLA2G4A : 1; C1orf99 : 1	Chromosome	Duplication	Heterozygous	Autosomal Recessive	G-banded chromoso me analysis
6.	Kim/South Korea/2020 [55]	CR	1	MFDM	EFTUD2 : 1	DNA	Deletion	Heterozygous	Autosomal Dominant	Sanger sequencing Minigene Assay
7.	Goldmuntz/Philadelphia/2011 [56]	CR	1	CFM	NRP1 : 1	DNA	Deletion	Heterozygous	Autosomal Dominant	PCR
8.	Brun/France/2012 [57]	CR	1	15q24 deletion Syndrome, OAVS, CFM	STRA6 : 1; and other unexplained 36 genes involved	Chromosome	Deletion	Heterozygous	Autosomal Recessive	Array CGH
9.	Koprulu/Turkey/2021 [58]	CR	1	FRASRS1: 1	GRIP1 : 1	DNA	Deletion	Homozygous	Autosomal Recessive	Sanger sequencing
10.	Hu/America/2019 [59]	CR	1	-	FGFR1 : 1	DNA	-	Heterozygous	Autosomal Dominant	NGS
11.	Jarzabek/Poland/2012 [60]	CR	1	Kallmann Syndrome: 1	FGFR1 : 1	DNA	-	Homozygous	Autosomal Dominant	Sanger technique
12.	Knapp/Maryland/202 0 [61]	CR	1	MGORS: 1	DONSON : 1	DNA	-	Heterozygous	Autosomal Recessive	Chromium WGS sequencing
13.	Saviola/Italy/2021 [62]	CR	1	CSS1: 1	ARID1A : 1	DNA	-	Heterozygous	Autosomal Dominant	NGS and PCR
14.	Lacour/New Orleans/2018 [63]	CR	1	MFDM:1 CFM: 1	EFTUD2 : 1	DNA	-	Heterozygous	Autosomal Dominant	WES
15.	Bragagnolo/Brazil/2016 [64]	CR	1	WHS: 1	-	Chromosome (4p-deletion syndrome)	Deletion	Heterozygous	Autosomal Dominant	FISH, CGH, PCR
16.	Knapp/New Zealand/20201 [43]	CR	1	MGORS: 1	CDT1 : 1	RNA	Deletion	Heterozygous	Autosomal Recessive	PCR
17.	Liu/China/2021 [15]	CR	1	TCS: 1	TCOF1 : 1	DNA	Nonsense Mutation	Heterozygous	Autosomal Dominant	WES Sureselect XT Target Enrichment system, Microarray, Sanger Sequencing
18.	Maya/Israel/2020 [65]	CR	1	CCA: 1	-	Chromosome	Deletion	Heterozygous	Autosomal Dominant	MicroArray
19.	Okamoto/Japan/2022 [21]	CS	2	-	MARS1 : 2	RNA : 2	Missense : 2	Heterozygous	Autosomal Recessive	WES Sanger sequencing
20.	Brophy/Iowa USA/2013 [66]	CS	3	BOR: 3	EYA1 : 1	Chromosome: 3	Deletion : 2 Duplication:1	Heterozygous	Autosomal Dominant	Array Based CGH
21.	Bukowska/Poland/2020 [22]	CS	3	TCS : 3	TCOF1 : 3	DNA : 3	Duplication: 2 Deletion: 1	Heterozygous	Autosomal Dominant	Sanger Sequencing
22.	Tingaud- Sequeira/France/2021 [67]	CS	2	Goldenhar Syndrome: 2 CFM: 1	EYA3 : 2	DNA : 2	Missense : 2	Heterozygous	Autosomal Dominant	WES
23.	Kim/South Korea/2017 [68]	CS	2	-	CDT1 : 2	DNA : 2	Duplication: 2	Heterozygous	Autosomal Recessive	Sanger sequencing
24.	Jung/New York/2020 [34]	CS	4	-	FANCB variant: 4	DNA : 4	Large deletion: 1 Insertion & Deletion: 1 Missense:1 Nonsense: 1	Heterozygous	X-linked Recessive	RT-PCR
25.	Su/Taiwan/2007 [14]	CS	5	OAVS: 4 TCS: 1 CFM: 3	TCOF1 : 5	DNA : 4	Silent mutation: 2	Heterozygous	Autosomal Dominant	PCR
26.	Chen/China/2017 [17]	CS	19	TCS: 19	TCOF1 : 19	DNA : 18	Deletion: 11 Insertion: 1 Missense: 2	Heterozygous	Autosomal Dominant	PCR, Sanger Technique
27.	Royer- Bertrand/Switzerland/ 2015 [20]	CS	3	EVPLS: 3	HSPA9 : 3	DNA : 3	-	Homozygous	Autosomal Recessive	Sanger sequencing
28.	Zhang/China/2016 [69]	CH	984	984 CFM, unspecifie d	ART3 : 1; ASB18 : 1; C15orf39 : 1; CCDC33 : 1; CSK : 1; CSPG4 : 1; CYP11A1 : 1; DBF4B : 1; FMNL1 : 1; FRMD4A : 1; FRMD6 : 1; GATA3 : 1; GFAP : 1; GJC1: 1; GOLGA6A : 1; HEXIM2 : 1; IGHMBP2 : 1; ISLR : 1; ISLR2 : 1; KLF12 : 1; LOC10099651 5 : 1; MYEOV: 1; NPAP1 : 1; PLCD3 : 1; PML : 1; PRKCE : 1; SCARB2 : 1; SHROOM3 : 1; SOCS5 : 1; SRBD1 : 1; TMEM247 : 1	-	Missense: 29 Frameshift: 2	Heterozygous	Autosomal Dominant	PCR
29.	Monks/United Kingdom/2010 [70]	CC	12	- (isolated microtia)	SIX2 : 3 HOXA2 : 2	DNA : 5	-	Heterozygous	SIX2 : Autosomal Dominant HOXA2 : Autosomal Recessive	MassARRAY Assay
30.	Zhang/China/2009 [31]	CC	121	- (isolated microtia)	Gsc Exon 2 : 6 Gsc Exon 3 : 2 BMP 5 maternal peptide : 1	DNA: 8 RNA: 1	Silent mutation: 6 Missense: 3	Heterozygous	Gsc Exon 2&3: Autosomal Recessive BMP 5 maternal peptide : Autosomal Dominant	Direct sequencing

*Abbreviations*: *TCS, MIM#154500* Treacher-Collins Syndrome, *CFM, MIM#164210* Craniofacial Microsomia, *MFDM, MIM#610536* Mandibulofacial Dysostosis with Microcephaly, *OAVS, MIM#164210* Oculo-auriculo-vertebral spectrum, *MGORS1, MIM#224690* Meier- Gorlin Syndrome, *WHS, MIM#194190* Wolf-Hirschhorn Syndrome, *BOR, MIM#113650* Branchio-oto-renal syndrome, *EVPLS, MIM#616854* Even-plus Syndrome, *WITKOS, MIM#613406* 15q24 deletion Syndrome / Witteveen Kolk Syndrome, *FRASRS1, MIM#219000* Fraser syndrome, *MIM 308750* Kalmann Syndrome, *CSS1, MIM#135900* Coffin-Siris Syndrome. *CCA, MIM#121050* Beals Syndrome, *CR* Case report, *CS* Case series, *CH* Cohort, *CC* Case-control

**Table 3:** Patient sex characteristics (*n=* 1495)

**SEX**	**N**	**%**
Male	186	13%
Female	80	5%
Unknown	1193	82%
**TOTAL**	**1459**	**100%**

**Fig. 3:**
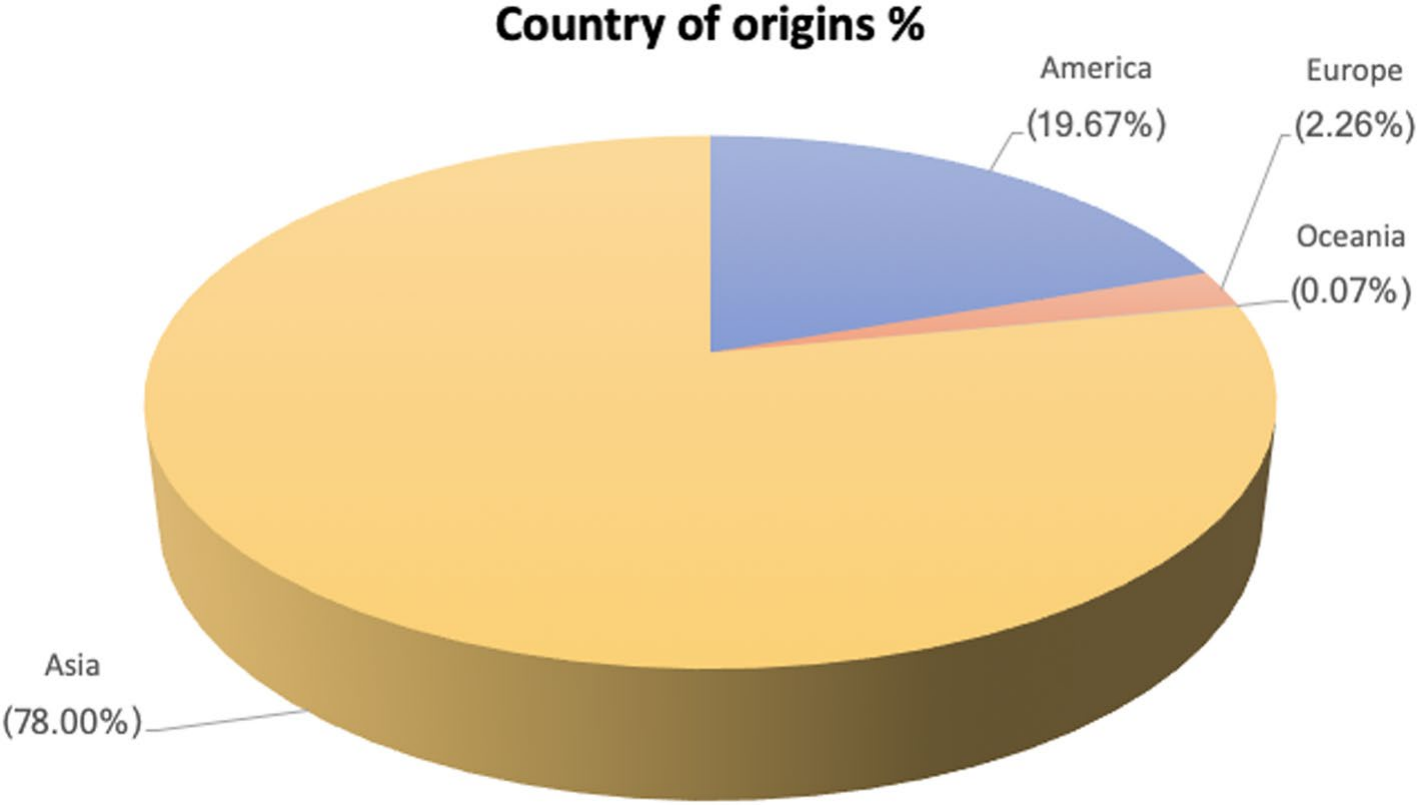
Demographics of origin of patients stratified by continent (*n=* 1459) (basic continent classifications refer to the UNSD Methodology) [72]

We classify the continents based on the UNSD Methodology [71]. Most cases are distributed in Asia (78.00%), America (19.67%), followed by Europe (2.26%) (Fig. 3). Of 1459 cases, there are 133 (9.12%) isolated microtia or non-syndromic cases, while syndromic microtia has 1244 (85.26%) cases, and 82 (5.62%) cases with unclear syndrome descriptions. A total of 1159 cases with microtia-affected sites or syndromic cases were reported. We found microtia on the right ear (64.5%) and the left side (35.4%) in unilateral side microtia.

Microtia grade III is the highest grade we found in this study, with 782 (71.29%) of 1097 cases, followed by grade II (24.25%), grade I (2.28%), and grade IV (2.19%). From the phenotype report, we found 1257 syndromes related to microtia, including 1191 cases of CFM (94.75%), 29 cases of TCS (2.31%), 9 cases of Oculo-auricular-vertebral spectrum (OAVS) counted (0.72%), 8 cases Branchiootorenal syndrome (BOR, MIM #113650) (0.64%) (Table 4).

**Table 4:** Common syndromes reported in microtia subject

**SYNDROME**	**n**	**%**
**All Syndromes Found**	**1257**	**100.00%**
Craniofacial Microsomia	1191	94.75%
Treacher Collin Syndrome	29	2.31%
Branchiootorenal Syndrome	8	0.64%
OAVS	9	0.72%
Goldenhar Syndrome	5	0.40%
Meier Gorlin Syndrome	2	0.16%
Mandibulofacial dysostosis with microcephaly Syndrome	2	0.16%
Wolf-Hirschhorn Syndrome	1	0.08%
Coffin-siris Syndrome	1	0.08%
15q24 deletion Syndrome	1	0.08%
Kalmann Syndrome	1	0.08%
Fraser Syndrome	1	0.08%
Cat Eye Syndrome	1	0.08%
Beals Syndrome	1	0.08%
trisomy 13 mosaicism	1	0.08%
EVEN-PLUS	3	0.24%

**Fig. 4:**
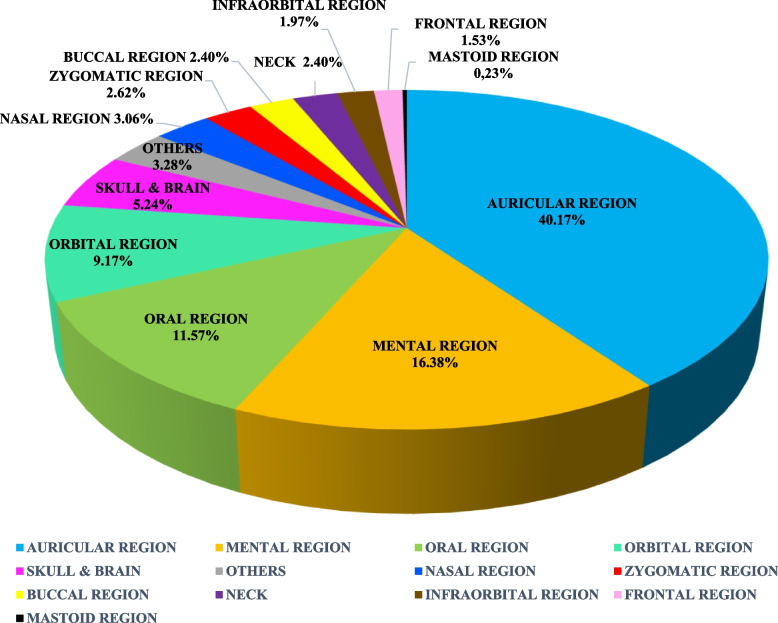
Other phenotypes accompany microtia in the head and neck region

Besides microtia, there are also accompanying phenotypes found. We found 458 phenotypes in the head and neck region beside microtia, including atresia of the external auditory canal in 63 cases (13.76%), mandibular hypoplasia (12.45%), preauricular tags (6.11%), and others (Table 5). Other accompanying phenotypes are grouped based on head and neck regions [73] (Fig. 4).

**Table 5:** Phenotypes characteristics in head & neck region

**OTHER PHENOTYPES**	**n**	**%**
**All Other Phenotypes Found**	**458**	**100%**
**HEAD**		
**ORBITAL REGION**		
Downward slanting palpebral fissures	9	1.97%
Eyelid coloboma	6	1.31%
Epicanthic folds	4	0.87%
Partial Absent of eyelashes	4	0.87%
Hypertelorism	4	0.87%
Arched & synophrys eyebrows	3	0.66%
Absence of eyelid	2	0.44%
Posterior Embryotoxon	1	0.22%
Orbital hypoplasia	1	0.22%
Broad Medial eyebrow	1	0.22%
Exophtalmia	1	0.22%
Deep-set eyes	1	0.22%
Optic Nerve hypoplasia	1	0.22%
Underdeveloped supraorbital ridge	1	0.22%
Ptosis	1	0.22%
Microphtalmia	1	0.22%
Anopthalmia	1	0.22%
**INFRAORBITAL REGION**		
Maxillary hyp.	6	1.31%
Prominent maxilla with overbite and malocclusionup	1	0.22%
Hypertrofi maxillary sinus	1	0.22%
Midface hypoplasia	1	0.22%
**ZYGOMATIC REGION**		
Zygoma / Malar hypoplasia	12	2.62%
**BUCCAL REGION**		
Facial cleft	10	2.18%
Cheek soft tissue tags	1	0.22%
**NASAL REGION**		
Flat nose	3	0.66%
Triangular nostrils	3	0.66%
Prominent Nose	2	0.44%
Bulbous nose	2	0.44%
Narrow nose	2	0.44%
High nasal bridge	2	0.44%
**ORAL REGION**		
High-arched palate	11	2.40%
Cleft lip & palate	10	2.18%
Cleft palate	9	1.97%
Narrowed Palate	3	0.66%
Microstomia	3	0.66%
Right-sided palatoplegia	2	0.44%
Small mouth with full lips	2	0.44%
Macrostomia	2	0.44%
Cleft lip only	1	0.22%
Tented lip	1	0.22%
Fissure in the alveolar crest	1	0.22%
Per incisors are overjet	1	0.22%
Diastema, dental crowding, and unerupted teeth	1	0.22%
Downward Slant of mouth right side	1	0.22%
Bifid Uvula	1	0.22%
Thin upper Lip	1	0.22%
Full Bottom Lip	1	0.22%
Anterior Open Bite	1	0.22%
Hypodontia	1	0.22%
**MENTAL REGION**		
Mandibular hyp.	57	12.45%
Micrognathia	12	2.62%
Retrusive Mandibular	3	0.66%
Retrognathia	1	0.22%
Prognathism	1	0.22%
Narrow face	1	0.22%
**AURICULAR REGION**		
External auditory canal atresia	63	13.76%
Preauricular tags	28	6.11%
Hearing loss	24	5.24%
External auditory canal stenosis	19	4.15%
Middle Ear hypoplasia	10	2.18%
Dysplasia M. incudal and Stapes-incudal articulations	9	1.97%
Dysplastic Ear	9	1.97%
Low set ears	8	1.75%
Preauricular pits	4	0.87%
Cup Ear deformity	3	0.66%
Reduced tympanic volume	2	0.44%
Cholesteatoma	2	0.44%
Granulomatous eardrum and the mastoid region	1	0.22%
Fistula of ears	1	0.22%
Multiple pinna on one side	1	0.22%
**FRONTAL**		
Narrowed frontal bone	2	0.44%
Forehead retrusion	1	0.22%
High anterior hairline	1	0.22%
Sloping forehead	1	0.22%
High Forehead	1	0.22%
Hypertrophied frontal sinuses	1	0.22%
**MASTOID**		
Hypoplasia Mastoid Complex	1	0.22%
**SKULL & BRAIN**		
Microcephaly	5	1.09%
Hydrocephalus	3	0.66%
Aplasia cutis on the skull	2	0.44%
Dolichocephaly	2	0.44%
Semilobar HPE	1	0.22%
Corpus Callosum Dysgenesis	1	0.22%
Frontal Periventricular Gliosis	1	0.22%
Metopic craniosynostosis	1	0.22%
Trigonocephaly	1	0.22%
Aplasia cranial fossa floor	1	0.22%
Cerebral hypoplasia	1	0.22%
Ventricular dilatation	1	0.22%
Incomplete Closure of the anterior fontanelle	1	0.22%
Persistent open anterior fontanelle	1	0.22%
Two lateral hair whorls	1	0.22%
Brachycephaly	1	0.22%
**NECK**		
Tracheoesophageal fistula	3	0.66%
Tracheal Stenosis & malacia	2	0.44%
Branchial Fistulae	1	0.22%
Branchial Tag	1	0.22%
Hypersegmented Cervical Vertebrae	1	0.22%
Hashimoto Thyroiditis	1	0.22%
Esophageal atresia	1	0.22%
Short neck	1	0.22%
**OTHERS**		
Dermoid	8	1.75%
Facial implantation of the hair	3	0.66%
Intellectual disability	2	0.44%
Speech delayed	2	0.44%

**Fig. 5:**
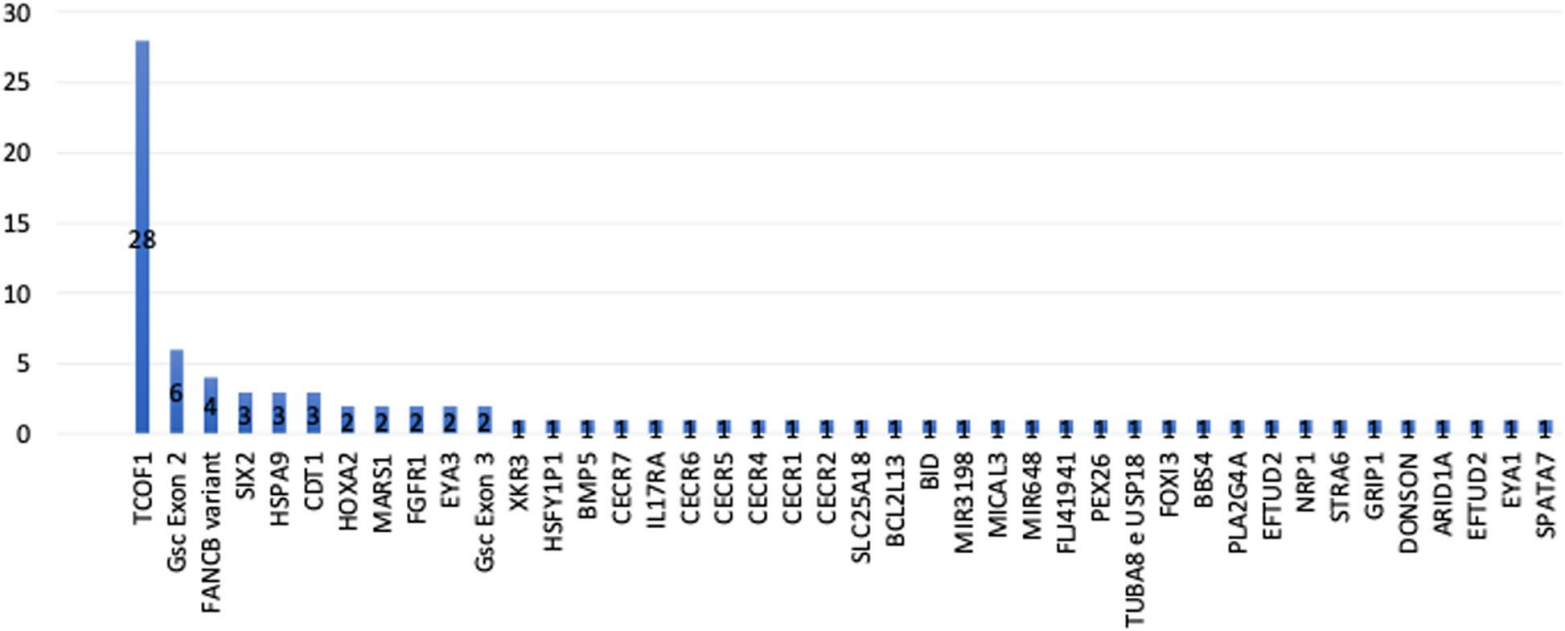
All genes involved in the occurrence of microtia

In total, there are 73 levels of genetic disorders reported. The highest level of genetic disorders is 59 (80.82%) DNA, followed by chromosome 10 (13.70%), then RNA 4 (5.48%). Missense (52.63%) is the most common mutation type we found in all genes reported here, followed by deletion (31.58%) and silent mutation (11.48%) (Table 6). As reported in this review, the major gene disorder related to microtia phenotype is found in *TCOF1* (32.82%; 28 cases), followed by *GSC* exon 2 (6.82%), *FANCB* (4.55%), *SIX2* (3.41%), *HSPA9* (3.41%) and *CDT1* (3.41%) (Fig. 5) with each characteristic (Table 7). Several variant types were found on *TCOF1*, the major gene in this review. Deletion (42,85%) is the most common type of variant, followed by missense (7.14%), duplication (7.14%), and silent (7.14%). Six patients had a family history of microtia. In these patients, the *MARS1* gene was found in two (33.33%) patients who were siblings, *TCOF1* was found in two other patients who stated that they were one family, and *HSPA9* in two patients from other families who were siblings.

**Table 6:** Variant types of all genes

**MUTATION TYPE**	**n**	**%**
Missense	40	52,63%
Deletion	24	31,58%
Silent Mutation	9	11,84%
Frameshift	2	2,63%
Nonsense	1	1,32%
**TOTAL**	**76**

**Table 7:** Characteristic of genes involved with microtia

**Gene**	**n**	**%**
*TCOF1*	28	31.82%
*GSC Exon 2*	6	6.82%
*FANCB*	4	4.55%
*SIX2*	3	3.41%
*HSPA9*	**3**	3.41%
*CDT1*	3	3.41%
*HOXA2*	2	2.27%
*FGFR1*	2	2.27%
*EYA3*	2	2.27%
*MARS1*	2	2.27%
*GSC Exon 3*	2	2.27%
*XKR3*	1	1.14%
*HSFY1P1*	1	1.14%
*GAB4*	1	1.14%
*CECR7*	1	1.14%
*IL17RA*	1	1.14%
*CECR6*	1	1.14%
*CECR5*	1	1.14%
*CECR4*	1	1.14%
*CECR1*	1	1.14%
*CECR2*	1	1.14%
*SLC25A18*	1	1.14%
*BCL2L13*	1	1.14%
*BID*	1	1.14%
*MIR3198*	1	1.14%
*MICAL3*	1	1.14%
*MIR648*	1	1.14%
*FLJ41941*	1	1.14%
*PEX26*	1	1.14%
*TUBA8 e USP18*	1	1.14%
*FOXI3*	1	1.14%
*BBS4*	1	1.14%
*PLA2G4A & C1orf99*	1	1.14%
*EFTUD2*	1	1.14%
*NRP1*	1	1.14%
*STRA6*	1	1.14%
*GRIP1*	1	1.14%
*DONSON*	1	1.14%
*ARID1A*	1	1.14%
*EFTUD2*	1	1.14%
*EYA1*	1	1.14%
*SPATA7*	1	1.14%
**TOTAL**	**88**	**100%**

Three patients had more than one gene abnormality. The first patient with phenotypic abnormalities of Cat Eye Syndrome (CES, MIM #115470), OAVS, and CFM had more than one responsible gene: *BCL2L13, BID, CECR1-CECR7, FLJ41941, GAB4, HSFY1P1, IL17RA, MICAL3, MIR3198, MIR648, PEX26, SLC25A18, TUBA8e, USP18*, and *XKR3*. The second patient with phenotypic abnormalities of CFM syndrome showed variants in *PLA2G4A* and *C1orf99* genes. The third patient with 15q24 deletion syndrome, OAVS, and CFM revealed responsible genes of *STRA6* and 36 other unexplained genes. Notably, these genes are not the majority in our study.

We grouped microtia patients with genetic data based on whether the patients had syndromic or non-syndromic microtia. Of the 88 patients with genetic data, 64 (72.72%) had syndromic microtia (Table 8), and 24 (27.27%) had non-syndromic microtia. In the syndromic microtia group, the most common genes were *TCOF1* (43.75%; 28 out of 64 cases), *SIX2* (4.69%), and *HSPA9* in (4.69%) patients. In the non-syndromic microtia group, the most frequently found gene was *GSC* exon 2 (25%; 6) and *FANCB* (16.67%); *HOXA2*, *GSC* exon 3, *MARS1*, *CDT1* were found respectively in two (8.33%) cases (Table 9). CFM syndromes have the most common genes involved (Table 10).

**Table 8:** Syndromes reported in genetics articles

**Syndrome**	**n**	**%**
**All Syndromes Found**	**1043**	**100%**
CFM	993	95,21%
TCS	25	2,40%
OAVS	6	0,58%
BOR Syndrome	3	0,29%
EVEN-PLUS	3	0,29%
MGORS	2	0,19%
Goldenhar Syndrome	2	0,19%
MFDM	2	0,19%
15q24 deletion Syndrome	1	0,10%
Coffin-Siris Syndrome	1	0,10%
WHS	1	0,10%
Beals Syndrome	1	0,10%
Fraser Syndrome	1	0,10%
Kallmann Syndrome	1	0,10%
CES	1	0,10%

**Table 9:** Genes related syndromic and non-syndromic microtia

**Non-Syndromic Genes**	**n**	**%**
Gsc Exon 2	6	25.00%
FANCB variant	4	16.67%
HOXA2	2	8.33%
Gsc Exon 3	2	8.33%
MARS1	2	8.33%
CDT1	2	8.33%
FGFR1	1	4.17%
NRP1	1	4.17%
FOXI3	1	4.17%
BBS4	1	4.17%
PLA2G4A & C1orf99	1	4.17%
BMP 5	1	4.17%
**Total**	**24**	**100.00%**
**Syndromic Microtia Genes**	**n**	**%**
TCOF1	28	43.75%
SIX2	3	4.69%
HSPA9	3	4.69%
EYA3	2	3.13%
FGFR1	1	1.56%
EYA1	1	1.56%
SPATA7	1	1.56%
XKR3	1	1.56%
HSFY1P1	1	1.56%
GAB4	1	1.56%
CECR7	1	1.56%
IL17RA	1	1.56%
CECR6	1	1.56%
CECR5	1	1.56%
CECR4	1	1.56%
CECR1	1	1.56%
CECR2	1	1.56%
SLC25A18	1	1.56%
BCL2L13	1	1.56%
MIR3198	1	1.56%
MICAL3	1	1.56%
MIR648	1	1.56%
FLJ41941	1	1.56%
PEX26	1	1.56%
TUBA8 e USP18	1	1.56%
EFTUD2	1	1.56%
STRA6	1	1.56%
GRIP1	1	1.56%
DONSON	1	1.56%
ARID1A	1	1.56%
EFTUD2	1	1.56%
CDT1	1	1.56%
**Total**	**64**	**100.00%**

**Table 10:** Syndromes and related genes

**Syndrome**	**Related Genes**	**n**
TCS (*n=* 25)	*TCOF1*	24
	*SPATA7*	1
CFM (*n=* 9)	*TCOF1*	3
	*C1orf99*	1
	*NRP1*	1
	*STRA6*	1
	*CYP11A1*	1
	*MPI*	1
	*EFTUD2*	1
	*EYA3*	1
	*PLA2G4A*	1
	*ATP6V1E1*	1
	*BCL2L13*	1
	*BID*	1
	*CECR1*	1
	*CECR2*	1
	*CECR4*	1
	*CECR5*	1
	*CECR6*	1
	*CECR7*	1
	*FLJ41941*	1
	*GAB4*	1
	*HSFY1P1*	1
	*IL17RA*	1
	*MICAL3*	1
	*MIR3198*	1
	*MIR648*	1
	*PEX26*	1
	*SLC25A18*	1
	*TUBA8 e*	1
	*USP18*	1
	*XKR3*	1
OAVS (*n=*6)	*TCOF1*	4
	*ATP6V1E1*	1
	*BCL2L13*	1
	*BID*	1
	*CECR1*	1
	*CECR2*	1
	*CECR4*	1
	*CECR5*	1
	*CECR6*	1
	*CECR7*	1
	*FLJ41941*	1
	*GAB4*	1
	*HSFY1P1*	1
	*IL17RA*	1
	*MICAL3*	1
	*MIR3198*	1
	*MIR648*	1
	*PEX26*	1
	*SLC25A18*	1
	*TUBA8 e*	1
	*USP18*	1
	*XKR3*	1
	*STRA6*	1
	*CYP11A1*	1
	*MPI*	1
BOR Syndrome (*n=* 3)	*EYA1*	1
EVEN-PLUS (*n=* 3)	*HSPA9*	3
Goldenhar Syndrome (*n=* 2)	*EYA3*	2
MDFM Syndrome (*n=* 2)	*EFTUD2*	2
MGS (*n=* 2)	*DONSON*	1
	*CDTI*	1
CES (*n=* 1)	*ATP6V1E1*	1
	*BCL2L13*	1
	*BID*	1
	*CECR1*	1
	*CECR2*	1
	*CECR4*	1
	*CECR5*	1
	*CECR6*	1
	*CECR7*	1
	*FLJ41941*	1
	*GAB4*	1
	*HSFY1P1*	1
	*IL17RA*	1
	*MICAL3*	1
	*MIR3198*	1
	*MIR648*	1
	*PEX26*	1
	*SLC25A18*	1
	*TUBA8 e*	1
	*USP18*	1
	*XKR3*	1
	*CECR7*	1
	*FLJ41941*	1
	*GAB4*	1
	*HSFY1P1*	1
	*IL17RA*	1
	*MICAL3*	1
	*MIR3198*	1
	*MIR648*	1
	*PEX26*	1
	*SLC25A18*	1
	*TUBA8 e*	1
	*USP18*	1
	*XKR3*	1
15q24 deletion syndrome (*n=* 1)	*STRA6*	1
	*CYP11A1*	1
	*MPI*	1
Frasser Syndrome (*n=* 1)	*GRIP1*	1
Kalmann Syndrome (*n=* 1)	*FGFR1*	1
Coffin-Siris Syndrome (*n=* 1)	*ARID1A*	1

## Discussion

In this study, we aimed to identify the genes associated with microtia, associated syndromes, and the presence of other phenotypic abnormalities in the head and neck region that are currently poorly understood. Based on our demographic characteristics data, Asia had the highest number of microtia cases in this investigation. This study's findings align with epidemiological data provided by a previous study [72], which states that Asian descent has a higher prevalence of microtia [72]. This study compared 186 male microtia patients to 80 female patients. The rest of the data needed to be clarified. The sex ratio in this study found that more microtia occurred in males, similar to the previous studies [1, 8, 74]. The ratio found in this study was 2.3:1.

We found 1029 (88.78%) cases of unilateral microtia; bilateral microtia was only found in one out of ten patients. This finding was consistent with a previous study that showed microtia was most common on the unilateral side, with bilateral microtia present in 2 out of 10 patients [72]. The most common type of microtia in the literature is class III lobular microtia, which accounted for 71.29% of all cases in our investigation, in line with a previous study [5].

### Syndromes related to microtia

In this systematic review, there were 1244 cases (85.26%) of patients with associated syndromes and 133 (9.12%) of non-syndromic cases. We found that almost all cases associated with CFM were 94.75%, TCS 2.31%, and OAVS 0.72%. This result was in line with previous studies, which reported that 35-55% of microtia cases were associated with a syndrome [7] and commonly associated with OAVS, CFM, TCS, Nager Syndrome, and DiGeorge Syndrome (DGS, MIM #188400) [2].

CFM was the most found syndrome associated with microtia in this systematic review. CFM is a spectrum of malformations that primarily involves structures from the first and second branchial arches [11]. Therefore, its clinical features include facial asymmetry resulting from maxillary with or without mandibular hypoplasia, preauricular or facial tags, and ear malformations consisting of microtia, anotia, or aural atresia, hearing loss, and ocular abnormalities [12]. The most common phenotypes seen in the patients with CFM in our systematic review were mandibular hypoplasia (32.12%), external auditory canal atresia (26.67%), and preauricular tags (15.15%). A previous report showed that 39 patients with craniofacial microsomia found the most phenotypes were microtia (75%) and facial hypoplasia (52%), followed by various types of tags (46%) [13]. According to our findings, the most common gene seen in CFM patients was *TCOF1*. *TCOF1* has been studied as a gene that has a role in the development of craniofacial anomalies related to CFM and also strongly associated with TCS [14].

We also found in our review that the *TCOF1* gene was most commonly found in microtia patients with TCS. *TCOF1* is an autosomal dominant mode of inheritance gene and is the major gene involved in TCS [15, 16]. TCS is a rare congenital disorder characterized by malformations of the bilateral middle and lower facial bones, coloboma of the lower eyelid, and external and middle ear malformation associated with bilateral conductive hearing loss [17]. In our review, the most common phenotypes associated in microtia patients with TCS were middle ear hypoplasia (9.85%), CHL (9.85%), and external auditory canal atresia (9.09%). Another study revealed that the most common phenotypes seen in patients with TCS were hypoplasia of the mandible, conductive deafness, and microtia [18].

Phenotypes in OAVS are variable, affecting the ears, eyes, face, neck, and other organs and systems. Minimum phenotypic inclusion criteria have yet to be agreed upon in the literature; however, the primary phenotype is hemifacial microsomia with facial asymmetry and microtia [16]. The most common head and neck phenotypes we found in this review are external auditory canal atresia (10.2%), incudal and stapes-incudal malleus articulation dysplasia (8.16%), Zygoma/malar hypoplasia (8.16%). This review found several genes involved with our OAVS patients, including *TCOF1, ATP6V1E1,* and *BCL2L13*. There have been hypotheses that the 22q11 genomic region and other genes are suspected of causing OAVS [19]. The three most common head and neck phenotypes in this study were external auditory canal atresia (13.76%), followed by mandibular hypoplasia (12.45%), and preauricular tags (6.11%). The results align with the most common phenotypes in each group of syndromes. The CFM group found that the most common phenotypes are mandibular hypoplasia, external auditory canal atresia, and preauricular tag. The group with OAVS found that the first typical phenotype was external auditory canal atresia.

### Genes related to microtia

We found 88 cases of genetic data related to microtia, including *TCOF1* (31.82%), *GSC* exon 2 (6.82%), *FANCB* (4.55%), *SIX2* (3.41%), *HSPA9* (3.41%), and *CDT1* (3.41%). This study showed different results from a previous study that found three genes most related to the development of microtia *HOXA2,* followed by *FGF3* and *TCOF1*, the third most common genes [5]. Based on our findings, 64 cases (72.72%) were syndromic microtia [*TCOF1* (43.75%), *SIX2* (4.69%), and *HSPA9* (4.69%)] and 24 cases (27.27%) were non-syndromic microtia [*GSC* exon 2 (25%), *FANCB* (16.67%), *HOXA2* (8.33%), *GSC* exon 3 (8.33%), *MARS1* (8.33%), *CDT1* (8.33%)]. In addition, *HSPA9*, *MARS1*, and *TCOF1* were the only genes related to familial microtia [20–22].

*The TCOF1* gene has been linked to more than 130 different variants. The variants observed so far arise throughout the gene, including missense, silent, insertion, duplication, deletion, splicing alterations, and nonsense variants. The most prevalent variants are deletions, which typically range in size from 1 to 40 nucleotides [23]. Most *TCOF1* variants cause loss of protein function and haploinsufficiency, with a predominantly autosomal dominant inheritance pattern [24]. Previous genetic, physical, and transcriptional mapping techniques identified that *TCOF1* was found to encode a low-complexity, serine/alanine-rich nucleolar phosphoprotein called Treacle protein. Treacle has a role in synthesizing ribosomal RNA, which helps the face's bones and cartilage to form [25]. A variant in the *TCOF1* gene will disrupt neural crest cell migration into the first arch during the fourth week of pregnancy [26], which can be called the first arch branchial syndrome [11]. The first arch branchial syndrome is a collection of congenital abnormalities involving the eyes, ears, mandible, and palate caused by abnormal first arch development. One example of the first arch branchial syndrome is TCS, which is strongly linked to a variant in the *TCOF1* [17]. Some *TCOF1* variants were functional single nucleotide polymorphisms (SNPs), including −948G>A, −1025G>C, and −346C>T, which have a frequency of more than 10% in public databases [27].

The homeobox protein goosecoid (*GSC*) is a homeobox protein gene [28]. This gene encodes a member of the bicoid subfamily of the paired (PRD) homeobox family of proteins that acts as a transcription factor and may be autoregulatory. These proteins act as a critical regulator during developmental processes in organogenesis, specifically the process of gastrulation in early embryonic development [29]. Animal studies have shown that variants in the *Gsc* have multiple defects of the lower mandible and the external auditory meatus [29, 30]. There are very few studies regarding variants in the *GSC* and their role in the development of microtia. This study found that the most common variant in the *GSC gene* was the silent variant (SNP) [29, 30], which involved *GSC exon 2* and *GSC exon 3* genes as non-syndromic microtia cases, such as 1244G>T [30, 31].

*FANCB* is a part of the Fanconi anemia complementation group (FANC). The *FANCB* gene product is the FANCB protein [32]. *FANCB* gene variants are X-linked recessive genes associated with Fanconi anemia. Most *FANCB* gene variants cause loss of protein function [33]. A previous study has also shown that individuals with *FANCB* variants have an earlier onset of bone marrow failure and more severe congenital anomalies than those without these variants [34]. Variants in the *FANCB* are highly associated with developing the VACTERL association. VACTERL is often associated with similar conditions, such as Goldenhar syndrome, including crossovers of conditions [35], which is known as OAVS [36]. In our review, we found that the phenotypes of *FANCB* were microcephaly, hydrocephalus, tracheoesophageal fistula, external auditory canal stenosis, esophageal fistula, and microphthalmia. There is no information on whether the Fanconi anemia patient is also associated with syndromes. A cohort study of 19 children with the deletion variant in *FANCB* demonstrated the earlier onset of bone marrow failure and more severe congenital abnormalities than those in the missense group [34]. We found bilateral microtia was only present in patients associated with deletion variants [34].

The *SIX2* gene is a family of *SIX* genes associated with the BOR syndrome, including external ear abnormalities and other congenital malformations [37]. The *SIX2* gene encodes homeobox protein SIX2 with an autosomal dominant pattern. It has recently been known as a set of transcription factors involved in embryonic morphogenesis renal causes Kidney and urinary tract abnormalities. During craniofacial development, it plays a role in the growth and elongation of the cranial base by regulating chondrocyte differentiation. It is seen as frontonasal dysplasia syndrome (FND, MIM #136760) and isolated microtia [38]. In line with our review, cases of isolated microtia in this study were found in 2 patients. Only variants in the *SIX2* gene were found in these patients, but no definite literature discusses isolated microtia and variants in *SIX2*. Isolated cases of microtia in *SIX2* variants may be related to loss of protein function and haploinsufficiency, which is associated with congenital ossicle malformation. The *SIX2* gene has been identified to be predominantly expressed in a large domain in the first branchial arch and a restricted one in the second branchial arch, so mutations in this gene can disrupt the process of ear formation. *SIX2* function will likely target general cartilage growth and differentiation regulators in the endochondral skeleton [39].

The *heat-shock 70 kDa protein nine* gene, also known as the *HSPA9*, has been understood to assist in protein folding, control cell proliferation, and inhibit apoptosis [40]. This gene has been shown to play a role in embryogenesis, cell movement, proliferation, morphogenesis, and apoptosis. In this review, variants of the *HSPA9* have been shown in this study to be recessive in the cases of EVEN-PLUS syndrome (EVPLS, MIM #616854) with microtia [20]_._

The *HOXA2* gene was found (8.33%) in this study as non-syndromic microtia cases. *HOXA2* is a transcription factor that plays a critical role in regulating embryonic development. Mutations in the *HOXA2* gene have been identified in individuals with microtia and associated craniofacial abnormalities. Most *HOXA2* variants cause loss of protein function [24]. These mutations disrupt the normal function of *HOXA2*, leading to disturbances in the development of ear structures during embryogenesis. Studies have shown that *HOXA2* is involved in the patterning and differentiation of the second branchial arch, giving rise to the outer and middle ear structures. Identifying the association between *HOXA2* variants and microtia provides essential insights into the genetic mechanisms underlying this condition [41]. Some *HOXA2* variants are SNPs, including g.90G>A and g.114A>C [30].

Based on our findings, 3 of 88 cases were related to the *Chromatin licensing and DNA replication factor 1* (*CDT1)* gene. One of them was a syndromic microtia case that was associated with Meier-Gorlin Syndrome (MGORS1, MIM #224690). In line with this, a study found that the CDT1 gene variants were related to Meier-Gorlin Syndrome patients with microtia phenotypes [42]. *CDT1* variants cause gain of function protein, with an autosomal recessive inheritance pattern that plays a vital role in DNA replication and cell cycle regulation, *CDT1* pre-replication complex mutation can disrupt the normal binding of CDT1 to its partner proteins, impairing its role in DNA replication and leading to abnormal ear development [43]. This study also found 2 cases of the *CDT1* gene as non-syndromic cases. However, the association between them is still unclear because there is still a lack of studies on non-syndromic microtia and *CDT1* genes.

*MARS1* (Methionyl-TRNA Synthetase 1) is a protein-coding gene that encodes the Methionyl-TRNA Synthetase 1 enzyme, which plays a vital role in protein synthesis by attaching the amino acid methionine to its corresponding tRNA molecule [44]. In this study, missense variants in the *MARS1* gene have been identified in individuals with microtia [21]. Most *MARS1* variants cause loss of protein function, with an autosomal recessive inheritance pattern. These variants disrupt the normal function of the methionyl-tRNA synthetase 1 enzyme, leading to impaired protein synthesis and subsequent abnormal translational insufficiency in specific stages of development, such as ear development [44]. Studies have highlighted the association between *MARS1* mutations and microtia, providing insights into the genetic mechanisms underlying this condition [21].

Nevertheless, *TCOF1* and *HOXA2*, in turn, cause microtia in a dominant manner, suggesting haploinsufficiency [24], while *HSPA9* and *GSC* are in recessive mode of inheritance [20, 29, 30]. In addition, there is no strong causative evidence referring to *SIX2* and isolated microtia.

Notably, the variable presentation observed in syndromic or non-syndromic microtia might also be ascribable to somatic mutations in genes that cause syndromes with auditory canal atresia and microtia. A previous study on twin studies supported the hypothesis that microtia might be due to a somatic variant that happens early in embryogenesis because monozygotic twins separate on day 12 following conception [45].

### Phenotypes in head and neck regions

Our study shows that more cases of microtia occur accompanied by other associated anomalies known as syndromic microtia (85.26%) cases. This anomaly mainly involves defects in the head and neck region caused by its embryological origins, both from the first and second pharyngeal arch.

The most common regions affected in this review were the auricular region 184 out of 440 (40.17%), with the most common phenotype reported being external auditory canal atresia. This data is relevant to the embryological processes of head and neck regions related to the pharyngeal arches, also known as branchial arches [26]. A temporary group of cells unique to vertebrates that arise from the embryonic ectoderm germ layer called Neural crest cells will migrate into the first pharyngeal arches to give rise to a diverse cell lineage [46]. In the case of microtia, various genetic and environmental factors can trigger the deregulation of cell-signaling pathways and disrupt neural crest cell migration, which can disrupt the pharyngeal arch, which in turn can cause different abnormalities in the formation [26].

This embryological process begins to occur in the fourth week, forming a maxillary prominence and a mandibular prominence [47]. Then, in the fifth week, the second pharyngeal arch will be overgrowth, resulting in an inward expansion of the first pharyngeal groove, forming the external acoustic meatus [48]. Furthermore, mesenchymal proliferation around the first and second pharyngeal arch, forming auricular hillocks, will further develop into the auricle [26]. The external auditory canal is derived from the first pharyngeal groove, the ectoderm, which undergoes inward expansion between the first and second pharyngeal arches. Therefore, if there was an abnormality in the pharyngeal arch, which afterward formed the external acoustic canal, it could cause abnormalities in the formation of the auricle [49].

The second most common region affected was the mental region (16.38%), with the most phenotype being mandibular hypoplasia. Suppose there is a disruption of migration of the neural crest in the first pharyngeal arches. In that case, it can disrupt the formation of the mandible and the auricle [26], which, as previously explained, may be due to the formation of both the mandible and the auricle associated with the same first pharyngeal arch.

The third most common phenotype is the oral region (11.57%), with the cleft lip and palate phenotype. The palate's formation process is formed from the primary and secondary palates, forming the definitive palate. The primary palate begins to develop in the sixth week by mesenchymal projection from medial nasal prominences. The secondary palate is formed in the sixth through the eighth weeks by the mesenchymal projection of maxillary prominence to the medial. Between the seventh and tenth week, there is a fusion of the medial nasal prominences with the maxillary and lateral nasal prominences, which is in time, by the twelfth week, the fusion of the nasal septum, primary and secondary palatine processes is completed [75]. This fusion will result in the continuity of the maxilla and upper lip and the separation of the nasal pits from the stomodeum as a primordium of the future mouth. The lower part of medial nasal prominences appears to have become deeply positioned and covered by the medial extension of the maxillary prominences to form the philtrum [26].

Syndromes associated with the pharyngeal arch can cause hypoplasia and aplasia along the structures formed by the related arch [26]. The most common patterns of malformations seen in patients with the syndrome in this study were TCS (94.75%) and CFM (2.31%) cases, which are thought to be caused by impaired development of structures derived from the first pharyngeal arches that occurred between the fifth and eighth week of embryonic development which is when the process of forming the head and neck is taking place. This may also be the basis for why most other phenotypic abnormalities occur in microtia in general in the head and neck region. Because the embryological processes of the head and neck regions are related, the earlier the disturbance occurs, the more regions will be affected and the more severe it will be.

## Limitations

This study has remaining limitations, such as the lack of observational studies that discuss the relation between the phenotype and genotype of microtia. Therefore, the studies included in this review are mostly case reports and case series. Some studies in this review also needed more data regarding their patients' phenotypes or genotypes. This systematic review also needed more data from a continent due to a lack of studies on microtia in that region.

## Conclusions

The most common accompanying phenotype of microtia patients was external ear canal atresia. The most common head and neck region abnormalities were the auricular, mental, and oral regions, which may be related to the embryological process associated with abnormalities of the first branchial arch that affect the embryological process of the three regions above. The most common syndrome found was CFM, with the most common phenotype being mandibular hypoplasia with the most common gene found being *TCOF1*. The three most common genes associated with microtia development were *TCOF1*, followed by *GSC* exon 2, *FANCB*, and an equal number of findings were *SIX2, HSPA9, and CDT1*. Most cases of microtia occurred in Asia, in line with other previous studies. Therefore, further observational studies with more complete and comprehensive data are needed, including patients with complete data on syndromes, phenotypes, and genotypes, especially in Asian populations.

### Supplementary Information

**Supplementary Material 1.**

**Supplementary Material 2.**

## Data Availability

The following supporting information can be downloaded at Harvard Dataverse: Genotype and Phenotype in Microtia (Supplementary Data). 10.7910/DVN/9AJN2A [76].
