# Supplementary material for: Genotype-phenotype associations in microtia: a systematic review
Source: Orphanet J Rare Dis. 2024 Apr 9;19:152. doi: 10.1186/s13023-024-03142-9 (PMC11003020; doi:10.1186/s13023-024-03142-9)
Supplement: Supplementary file 1 — Supplementary Material 1. [file 13023_2024_3142_MOESM1_ESM.docx]

Supplementary 1. Search keywords.

(("microtia"[Title/Abstract] OR "congenital microtia"[MeSH Terms] OR ("congenital microtia"[MeSH Terms] OR ("congenital"[All Fields] AND "microtia"[All Fields]) OR "congenital microtia"[All Fields] OR "microtia"[All Fields] OR "microtias"[All Fields]))) AND (((((("polymorphism, single nucleotide"[MeSH Terms] OR "polymorphism, single nucleotide"[MeSH Terms] OR "single nucleotide polymorphism*"[Title/Abstract] OR "polymorphism*"[Title/Abstract] OR "genotype*"[Title/Abstract] OR "varian*"[Title/Abstract] OR "mutation*"[Title/Abstract]) AND ((("trisomie"[All Fields] OR "trisomy"[MeSH Terms] OR "trisomy"[All Fields] OR "trisomies"[All Fields]) AND ("chromosom"[All Fields] OR "chromosomally"[All Fields] OR "chromosome s"[All Fields] OR "chromosomes"[MeSH Terms] OR "chromosomes"[All Fields] OR "chromosomal"[All Fields] OR "chromosome"[All Fields] OR "chromosomic"[All Fields] OR "chromosomically"[All Fields] OR "chromosomics"[All Fields]) AND "13"[All Fields]) OR (("trisomie"[All Fields] OR "trisomy"[MeSH Terms] OR "trisomy"[All Fields] OR "trisomies"[All Fields]) AND ("chromosom"[All Fields] OR "chromosomally"[All Fields] OR "chromosome s"[All Fields] OR "chromosomes"[MeSH Terms] OR "chromosomes"[All Fields] OR "chromosomal"[All Fields] OR "chromosome"[All Fields] OR "chromosomic"[All Fields] OR "chromosomically"[All Fields] OR "chromosomics"[All Fields]) AND "18"[All Fields]) OR "6p24"[All Fields] OR ("4p"[All Fields] AND ("duplicate"[All Fields] OR "duplicated"[All Fields] OR "duplicates"[All Fields] OR "duplicating"[All Fields] OR "duplication"[All Fields] OR "duplications"[All Fields] OR "duplicative"[All Fields])) OR "HOXA1"[All Fields] OR "HOXA2"[All Fields] OR "FGF3"[All Fields] OR "SIX1"[All Fields] OR "SIX3"[All Fields] OR "SIX4"[All Fields] OR "SIX5"[All Fields] OR "SIX2"[All Fields] OR "EYA1"[All Fields] OR "IRF6"[All Fields] OR "CHUK"[All Fields] OR "PRX1"[All Fields] OR "PRX2"[All Fields] OR ("green synth catal"[Journal] OR "glob stud child"[Journal] OR "gsc"[All Fields]) OR "PRKRA"[All Fields] OR ("thyroid cancer, papillary"[MeSH Terms] OR ("thyroid"[All Fields] AND "cancer"[All Fields] AND "papillary"[All Fields]) OR "papillary thyroid cancer"[All Fields] OR "pact"[All Fields]) OR "PLEC"[All Fields] OR "USH2A"[All Fields] OR "FREM2"[All Fields] OR "DCHS1"[All Fields] OR "GLI3"[All Fields] OR "POMT1"[All Fields] OR "GBA"[All Fields] OR "CHD7"[All Fields] OR "CDT1"[All Fields] OR "ORC1"[All Fields] OR "ORC4"[All Fields] OR "ORC6"[All Fields] OR "CDC6"[All Fields] OR "MED12"[All Fields] OR "TWIST1"[All Fields])) OR "hmx1"[All Fields] OR "tbx1"[All Fields])) OR ((((((human[MeSH Terms]) OR (genotype[MeSH Terms])) OR (mutation[MeSH Terms])) OR (phenotype[MeSH Terms])) OR (genetic*[MeSH Terms])) OR (dna copy number variatio*[MeSH Terms]))) OR (ear anomaly))
