# Supplementary material for: Genotype-phenotype associations in microtia: a systematic review
Source: Orphanet J Rare Dis. 2024 Apr 9;19:152. doi: 10.1186/s13023-024-03142-9 (PMC11003020; doi:10.1186/s13023-024-03142-9)
Supplement: Supplementary file 2 — Supplementary Material 2. [file 13023_2024_3142_MOESM2_ESM.docx]

#

Supplementary 2. JBI appraisal score.

**Note:**

# Y: YES, score 1 N: NO, score 0 U: U, score 0

**CASE REPORT**

Criteria

1. Were patient’s demographic characteristics clearly described?
2. Was the patient’s history clearly described and presented as a timeline?
3. Was the current clinical condition of the patient on presentation clearly described?
4. Were diagnostic tests or assessment methods and the results clearly described?
5. Was the intervention(s) or treatment procedure(s) clearly described?
6. Was the postNintervention clinical condition clearly described?
7. Were adverse events (harms) or unanticipated events identified and described?
8. Does the case report provide takeaway lessons?

| **First author’s surname/country/year** | **1** | **2** | **3** | **4** | **5** | **6** | **7** | **8** | **Score** |
| --- | --- | --- | --- | --- | --- | --- | --- | --- | --- |
| Gimelli/Italy/2013 | N | Y | Y | Y | Y | Y | N | Y | 6 |
| Glaeser/Brazil/2021 | Y | Y | Y | Y | Y | Y | N | Y | 7 |
| Tassano/Italy/2015 | N | Y | Y | Y | Y | Y | N | Y | 6 |
| Chaves/Brazil/2019 | Y | N | Y | Y | Y | Y | Y | Y | 7 |
| Huang/China/2013 | Y | Y | Y | Y | Y | Y | Y | Y | 8 |
| Kim/South Korea/2020 | Y | Y | Y | Y | Y | Y | Y | Y | 8 |
| Goldmuntz/Philadelpia/2011 | Y | N | Y | Y | Y | Y | N | Y | 6 |
| Brun/France/2012 | Y | Y | Y | Y | Y | Y | Y | Y | 8 |
| Koprulu/Turkey/2021 | Y | Y | Y | Y | Y | Y | N | Y | 7 |
| Hu/America/2019 | N | N | Y | Y | Y | Y | Y | Y | 6 |
| Jarzabek/Poland/2012 | N | Y | Y | Y | Y | Y | Y | Y | 7 |
| Knapp/ßMaryland/ | N | N | Y | Y | Y | Y | Y | Y | 6 |
| Saviola/Italy/2021 | Y | Y | Y | Y | Y | Y | Y | Y | 8 |
| Lacour/New Orleans/2018 | N | Y | Y | Y | Y | Y | Y | Y | 7 |
| Bragagnolo/Brazil/2016 | N | Y | Y | Y | Y | Y | Y | Y | 7 |
| Knapp/New Zealand/2021 | N | Y | Y | Y | Y | Y | N | Y | 6 |
| Liu/China/2021 | N | Y | Y | Y | Y | Y | Y | Y | 7 |
| Maya/Israel/2020 | Y | Y | Y | Y | Y | Y | N | Y | 7 |
| Chen/China/2017 | Y | Y | Y | Y | Y | N | Y | Y | 7 |
| DeGolovine/Texas/2012 | Y | Y | Y | Y | Y | Y | Y | Y | 8 |
| Griffith/Indianapolis/2009 | Y | Y | Y | Y | N | N | Y | Y | 6 |
| Lalani/Texas/2019 | Y | N | N | Y | Y | Y | Y | Y | 6 |

**CASE SERIES**

Criteria

1. Were there clear criteria for inclusion in the case series?
2. Was the condition measured in a standard, reliable way for all participants included in the case series?
3. Were valid methods used for identification of the condition for all participants included in the case series?
4. Did the case series have consecutive inclusion of participants?
5. Did the case series have complete inclusion of participants?
6. Was there clear reporting of the demographics of the participants in the study?
7. Was there clear reporting of clinical information of the participants?
8. Were the outcomes or follow up results of cases clearly reported?
9. Was there clear reporting of the presenting site(s)/clinic(s) demographic information?
10. Was statistical analysis appropriate?

| **First author’s surname/country/year** | **1** | **2** | **3** | **4** | **5** | **6** | **7** | **8** | **9** | **10** | **Score** |
| --- | --- | --- | --- | --- | --- | --- | --- | --- | --- | --- | --- |
| Okamoto/Japan/2022 | Y | Y | Y | N | N | Y | Y | Y | Y | U | 7 |
| Brophy/Iowa USA/2013 | Y | Y | Y | Y | N | Y | Y | Y | Y | N | 8 |
| Bukowska/Poland/2020 | Y | Y | Y | Y | N | N | Y | Y | Y | U | 7 |
| TingaudNSequeira/France/2021 | U | Y | Y | N | N | Y | Y | Y | Y | Y | 7 |
| Kim/South Korea/2017 | Y | Y | Y | Y | Y | Y | Y | Y | Y | U | 9 |
| Jung/New York/2020 | Y | Y | Y | Y | Y | N | Y | Y | Y | Y | 9 |
| Su/Taiwan/2007 | Y | Y | Y | N | N | Y | Y | Y | Y | U | 7 |
| Schmid/German/1985 | Y | Y | Y | Y | N | Y | Y | U | N | Y | 7 |
| Sutphen/Texas/1995 | Y | Y | Y | Y | Y | Y | Y | Y | Y | U | 9 |
| MartelliNJunior/Brazil/2009 | Y | Y | Y | Y | Y | Y | Y | Y | Y | U | 9 |
| Chen/China/2017 | Y | Y | Y | N | N | Y | Y | Y | Y | N | 7 |
| Patton/London/1995 | Y | N | N | Y | Y | Y | Y | Y | Y | N | 7 |

**CASE CONTROL**

Criteria

1. Were the groups comparable other than the presence of disease in cases or the absence of disease in controls?
2. Were cases and controls matched appropriately?
3. Were the same criteria used for identification of cases and controls?
4. Was exposure measured in a standard, valid and reliable way?
5. Was exposure measured in the same way for cases and controls?
6. Were confounding factors identified?
7. Were strategies to deal with confounding factors stated?
8. Were outcomes assessed in a standard, valid and reliable way for cases and controls?
9. Was the exposure period of interest long enough to be meaningful?
10. Was appropriate statistical analysis used?

| **First author’s surname/country/year** | **1** | **2** | **3** | **4** | **5** | **6** | **7** | **8** | **9** | **10** | **Score** |
| --- | --- | --- | --- | --- | --- | --- | --- | --- | --- | --- | --- |
| Luquetti/US/2015 | Y | Y | Y | Y | Y | N | N | Y | N | Y | 7 |
| Monks/UK/2010 | Y | Y | Y | Y | Y | N | N | Y | Y | Y | 8 |
| Zhang/China/2009 | Y | Y | Y | Y | Y | N | N | Y | N | Y | 7 |

**COHORT**

Criteria

1. Were the two groups similar and recruited from the same population?
2. Were the exposures measured similarly to assign people to both exposed and unexposed groups?
3. Was the exposure measured in a valid and reliable way?
4. Were confounding factors identified?
5. Were strategies to deal with confounding factors stated?
6. Were the groups/participants free of the outcome at the start of the study (or at the moment of exposure)?
7. Were the outcomes measured in a valid and reliable way?
8. Was the follow up time reported and sufficient to be long enough for outcomes to occur?
9. Was follow up complete, and if not, were the reasons to loss to follow up described and explored?
10. Were strategies to address incomplete follow up utilized?
11. Was appropriate statistical analysis used?

| **First author’s surname/country/year** | **1** | **2** | **3** | **4** | **5** | **6** | **7** | **8** | **9** | **10** | **11** | **Score** |
| --- | --- | --- | --- | --- | --- | --- | --- | --- | --- | --- | --- | --- |
| Heike/North America/2016 | Y | Y | Y | N | N | Y | Y | Y | Y | Y | Y | 9 |
| Luquetti/US/2019 | Y | Y | Y | N | N | N | Y | Y | Y | Y | Y | 8 |
| Zhang/China/2016 | Y | Y | Y | N | N | Y | Y | Y | Y | Y | Y | 9 |
| Lalani/Texas/2019 | Y | Y | Y | N | N | Y | Y | Y | Y | Y | Y | 9 |
